# Phenotypic Signatures Arising from Unbalanced Bacterial Growth

**DOI:** 10.1371/journal.pcbi.1003751

**Published:** 2014-08-07

**Authors:** Cheemeng Tan, Robert Phillip Smith, Ming-Chi Tsai, Russell Schwartz, Lingchong You

**Affiliations:** 1 Lane Center of Computational Biology, Carnegie Mellon University, Pittsburgh, Pennsylvania, United States of America; 2 Department of Biomedical Engineering, University of California Davis, Davis, California, United States of America; 3 Division of Mathematics, Science and Technology, Nova Southeastern University, Fort Lauderdale, Florida, United States of America; 4 Department of Biological Sciences, Carnegie Mellon University, Pittsburgh, Pennsylvania, United States of America; 5 Department of Biomedical Engineering, Duke University, Durham, North Carolina, United States of America; 6 Institute for Genome Sciences and Policy, Duke University, Durham, North Carolina, United States of America; National Center for Biotechnology Information (NCBI), United States of America

## Abstract

Fluctuations in the growth rate of a bacterial culture during unbalanced growth are generally considered undesirable in quantitative studies of bacterial physiology. Under well-controlled experimental conditions, however, these fluctuations are not random but instead reflect the interplay between intra-cellular networks underlying bacterial growth and the growth environment. Therefore, these fluctuations could be considered quantitative phenotypes of the bacteria under a specific growth condition. Here, we present a method to identify “phenotypic signatures” by time-frequency analysis of unbalanced growth curves measured with high temporal resolution. The signatures are then applied to differentiate amongst different bacterial strains or the same strain under different growth conditions, and to identify the essential architecture of the gene network underlying the observed growth dynamics. Our method has implications for both basic understanding of bacterial physiology and for the classification of bacterial strains.

## Author Summary

The measurement of bacterial growth in batch cultures is a routine practice in microbiology. In these cultures, bacterial growth rates drastically fluctuate over time due to the continuously changing growth environment: changing population size, accumulation of waste products, and depletion of nutrients. Such “unbalanced” growth is normally considered undesirable, which has led to the design of methods to achieve balanced growth environments (i.e., chemostats). However, we have discovered that unbalanced growth dynamics contain rich information that can be exploited to deduce regulatory functions or to classify cell strains or growth conditions. We further show that this approach is generally applicable to temporal gene expression data. Taken together, our method and results have broad implications for system identification, experimental design, and the study of cellular growth.

## Introduction

Bacterial growth is influenced by environmental factors, such as temperature, pH, and nutrient concentration [1]–[3]. When bacteria are grown in environments with limited nutrients, bacterial physiology can change considerably due to continually changing environmental factors. As a result of such “unbalanced” growth, the instantaneous growth rates can fluctuate drastically over time. These fluctuations are generally considered to be undesirable in quantitative studies of bacterial physiology and gene regulation. To limit these fluctuations, bacteria can instead be incubated in balanced growth environments so that bacterial physiology can be maintained at constant growth rates, which is typically achieved by using a chemostat or by periodic dilution of cultures [4]. Such growth environments, however, are neither natural nor readily amendable for high-throughput screening of bacterial physiology [5].

Instead, unbalanced growth is routinely used in high-throughput analyses of bacterial phenotypes and genotypes. For instance, knockout strains of *Escherichia coli* have been grown in batch cultures supplemented with different carbon and nitrogen sources. Here, the growth phenotypes of these bacterial strains were then used to construct a coupled metabolic and gene regulatory network [6]. Furthermore, *E. coli* strains from the Keio Collection have been previously grown on agar plates supplemented with different chemical stressors (i.e., drugs). From this analysis, the colony sizes of each bacterial strain were then used to cluster genes and drugs with similar functions [7]. Indeed, to increase the resolution of such unbalanced growth assays, several technologies have been used to study bacterial growth dynamics, including microfluidics [8], microscopes [9], and spectroscopy [10].

**Figure 1:**
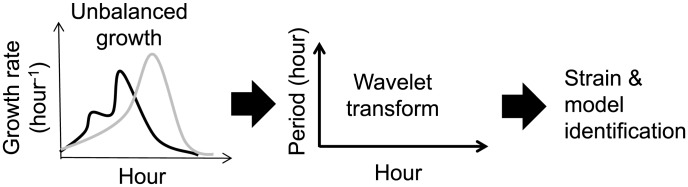
The analysis of unbalanced growth dynamics using wavelet transform. Unbalanced growth dynamics arise due to coupling between bacterial physiology and the growth environment (left panel). Such fluctuations are typically neglected in quantitative studies of bacterial growth but could be exploited as phenotype signatures using wavelet transform (middle panel). The signatures could be used to distinguish bacterial strains and infer growth models (right panel).

Under well-controlled experimental conditions, the fluctuations of growth rate during unbalanced growth are not random. Instead, they reflect the interplay between bacterial physiology and the growth environment: bacterial growth changes environmental conditions, which in turn influences bacterial growth. In particular, these fluctuations could be considered as quantitative phenotypes of bacteria under a specific growth condition (Figure 1). In this study, we refer to such fluctuations as cell-coupled perturbations.

The use of cell-coupled perturbations towards system identification is under-appreciated. In contrast, several studies have used controlled perturbations for system identification. For instance, previous studies have shown that complex [11] or multiple [12] input perturbations, as well as fluctuation resonance [13], can be used to improve the identification of reaction parameters. In addition, several studies have reconstructed cellular networks using cellular noise [14]–[16]. Finally, experimentally driven oscillatory stimuli have been used previously to reconstruct cellular pathways [17].

To identify systems using cell-coupled perturbations, we aimed to develop a set of empirical descriptors that capture growth dynamics by using wavelet transform [18]. Wavelet transform serves to decompose growth dynamics into time-frequency domains. Indeed, such decomposition has been used to analyze temporal dynamics [19]–[21] and to deduce dynamic signatures for system identification [22]. Furthermore, the decomposition can filter out noise from signals based on their frequencies. In this study, we take advantage of the decomposition to infer growth models using both modeling and experiments. Our results have implications for system identification, experimental design for cellular perturbations, and high-throughput bacterial phenotyping, with relevance for the diagnosis of infectious diseases [23].

## Results

**Figure 2:**
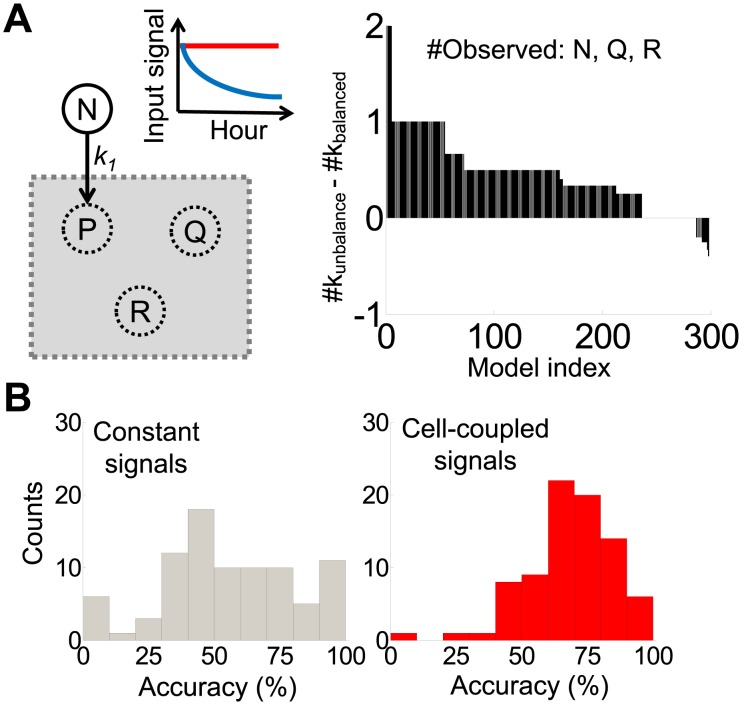
Unbalanced growth environments improve the identification of simple models. A. The difference of correctly estimated parameters between cell-coupled (k_unbalanced_) and constant signals (k_balanced_). Cell-coupled signals gave rise to overall higher parameter identifiability than constant signals. We created 500 models with random edges between three nodes (left panel, P, Q, R). We then compared parameter identifiability by using time series of N, Q, and R (right panel). Positive values indicate higher identifiability of parameters using cell-coupled signals. Negative values indicate higher identifiability using constant signals. The red line indicates time series of a constant input signal. The blue line represents time series of a cell-coupled signal. B. Histograms of the accuracy of estimated models using either constant or cell-coupled signals. Models estimated using cell-coupled signals (red bars) have a higher accuracy as compared to models estimated using constant signals (grey bars). Each histogram was calculated using 500 models.

It is well established that appropriate perturbations can enhance the identification of the system underlying experimental observations [11], [17], [24]. This notion has recently been applied to the analysis of gene regulation, where either controlled or endogenous perturbations (e.g., stochastic gene expression) have been exploited to identify gene circuits [11], [14]–[17], [25]. In the context of unbalanced growth, we hypothesized that the coupling between growth rate fluctuations and the changing growth environment would also facilitate system identification. To test this hypothesis, we constructed random minimal models to implement such coupling. Each model consists of four variables: three cellular components and an input signal (Figure 2A, node N) that represents a nutrient. While simple, these models can generate non-trivial dynamics with appropriate feedback loops between the cellular components [26].

To construct a minimal model, we generated six reaction links to connect the three cellular components randomly (nodes P, Q and R, Figure S1A) and generated random values for the system parameters. Node P was set to receive either a constant input flux or a cell-coupled input from the node N. We assumed that all nodes were observable and that all initial conditions were the same. Furthermore, we ensured that each of the links was not repeated. With each model, we simulated two distinct conditions by using either a constant or a cell-coupled signal. For each condition, we then used temporal dynamics of three simulated system variables to learn the system parameters. Using these models, we tested if coupling of an input signal to cellular components (cell-coupled signal) could generate more information about the underlying models than a constant and uncoupled input signal.

We used local parameter identifiability as a measure of information encoded in the system output [27]–[29]. This metric quantifies the likelihood of identifying an unknown parameter correctly using fluctuations of system variables. To accomplish this, we calculated local parameter identifiability by analyzing the algebraic structures of the transfer function matrix of each model [28], [29]. Briefly, we calculated a Jacobian matrix (*H*) of transfer functions (*g*) by differentiating *g* using each parameter. Next, we calculated the correlation matrix *R* of *H*. Our analysis generates a correlation matrix, where each element (−1≤*R_ij_*≤1) measures the correlation between two parameters (*k_i_* and *k_j_*). If *R_ij_* is close to 1 or −1, the two parameters are strongly correlated, which corresponds to low parameter identifiability (Text S1). Applying this method to an illustrative model (Figure S1A), we demonstrate that a cell-coupled signal enabled the identification of all parameters. With a constant signal, parameter *k_3_* was not identifiable. To test the generality of this observation, we generated 500 random models, each containing six random edges and randomized parameter values. For each model, we calculated the number of identifiable parameters by using either a cell-coupled or a constant signal. Our results show that cell-coupled signals significantly increased the number of identified parameters as compared to constant signals (Figure 2A).

We then investigated whether cell-coupled signals could improve the identification of unknown reaction links between system components in nonlinear models, which were generated using both mass action reactions and Michaelis-Menten reactions (Figure S1B). To emulate a constant-input environment, node N was set at a constant value. To emulate a cell-coupled input environment, we set node N to be consumed by node P (N→P in Figure 2). We simulated each “true” model using a random set of parameters. We assumed that all nodes are observable. The simulation results were augmented with 20% noise and used as the data to identify the models. We estimated linear models using a standard linear regression method [17], [30] (Equation 1 & Figure S1C & D).
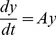
(1)where *y* represents the vector of molecular species, *t* represents time (hour), and *A* represents the reaction matrix. Briefly, we first used the regression method to generate a connectivity matrix *A* (Equation 1), where each element (*A_ij_*) specifies the likelihood that two variables are linked. We assumed they are linked when *A_ij_*>0.005. Increasing the *A_ij_* threshold to 0.01 does not change our conclusions. We then evaluated the accuracy of inference by using the true model as the reference. Specifically, each original model would have *m* “true” edges. Each estimated model would contain a subset *n* of these true edges. We calculated the ratio *n*/*m* as the metric of estimation accuracy. Based on the analysis of 500 random models, we found that cell-coupled signals significantly improved the accuracy of inference in comparison to constant signals (Figure 2B).

These two analyses show that cell-coupled signals indeed generate informative fluctuations in simple models that enhance system identification. We then hypothesized that this notion is applicable to the analysis of unbalanced bacterial growth. At a crude level, different strains of the same bacterial species may be governed by either the same cellular network with different parameters or similar networks with minor differences in cellular components and their interactions. A nutrient could be considered as a cell-coupled signal in a batch culture. Under the same initial growth condition, the distinct growth curves would then reflect the different dynamics of the underlying networks (and the associated parameters). Different strains can thus be classified according to their growth curves. Based on the simulation analysis described above, we hypothesized that unbalanced growth, in which cell-coupled signals lead to fluctuations of a system variable (growth rate), could improve such classification. Here, we used the growth rate as the measured system variable because it could be easily measured without introducing extrinsic reporters [31] and it most faithfully reflects the interplay between bacterial physiology and the growth environment [5].

**Figure 3:**
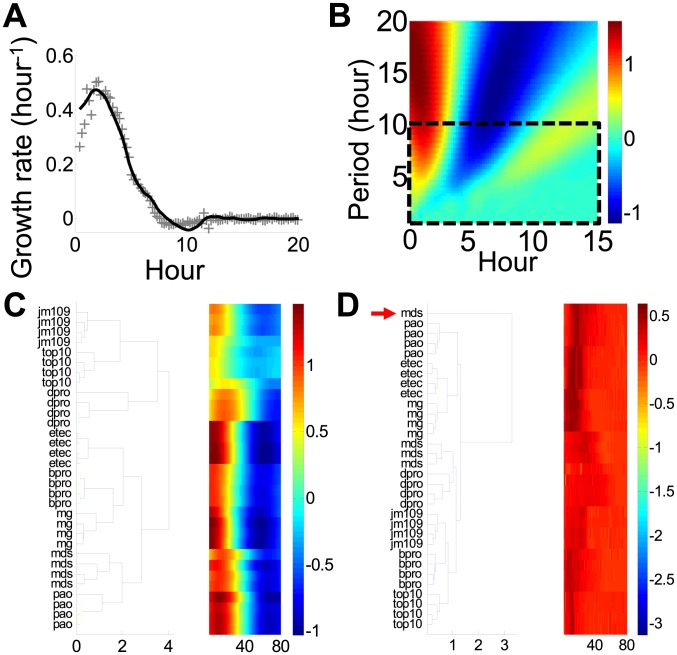
Unbalanced growth environments give rise to rich perturbations. A. A typical growth curve of MG1655z1 bacterial strain. Grey crosses represent original data. The black line represents the denoised growth curve using the “wden” function in Matlab with a Daubechies (db4) wavelet, a soft universal threshold and no rescaling. B. Wavelet transform of the raw growth curve (a) using a Daubechies (db4) wavelet. The heat map shows the amplitudes at each specific period and time-point. The black box indicates the range of periods that did not generate tight clusters of bacterial strains (Figure S2C). C. Classification of bacterial strains using the corresponding wavelet transforms. All bacterial strains were classified correctly. mg = MG1655z1, dpro = DH5αPro, pao = PAO1, mds = MDS42, bpro = BL21Pro, etec = ETEC, jm109 = JM109, top 10 = Top10. All data was classified using the standard hierarchical clustering algorithm in Matlab with the average Euclidean distance as the metric. D. Classification of bacterial strains using the raw growth curves. One strain was classified incorrectly, as indicated by the red arrow.

To test this idea, we collected high-temporal-resolution growth curves of six *Escherichia coli* strains, a *Pseudomonas aeruginosa* strain, and an enterotoxigenic *E. coli* (ETEC) strain (Table S1), all grown in batch cultures using M9 minimal media. Specific growth rates were calculated using the central differences of optical densities at each time point. For each growth curve, the specific growth rates fluctuated drastically over time (Figure 3A & Figure S2A). For comparison, we first calculated several conventional metrics, including maximal growth rates, final OD, and summation of differences (Table S2). Each of these metrics describes a specific aspect of a growth curve and is commonly reported in microbiology experiments [5], [32]. We found that each of these conventional metrics by itself could not distinguish the bacterial strains because mean values of each of these metrics were within one standard deviation for at least two of the strains. The fundamental limitation of these metrics is that they do not take full advantage of the rich temporal information in each growth curve, as reflected by the fluctuations in the specific growth rates. Therefore, all subsequent analyses were based on the entire growth curves.

We next transformed each time course of growth rates into the time-frequency domain, which was unique for each strain (Figure 3B & Figure S2B). All wavelet transform was performed using the Daubechies (db4) wavelet unless otherwise noted. The decomposition also separated random noise from signals by their frequencies. Based on the decomposition, informative empirical descriptors of growth dynamics could be extracted and used for system identification [22], [33]. We note that the advantages of wavelet transform have been exploited in the analysis of heart rhythm [19], the cell cycle [21], and metabolic pathways [20]. Here, we sought to determine the most informative wavelet frequencies that could distinguish different bacterial strains. To discover these frequencies, we used a hierarchical clustering method that classifies all growth curves into groups based on the average Euclidean distance between their wavelet coefficients. To assess the performance of the clustering method, we calculated the Davies–Bouldin score to assess the internal classification quality [34]. The Davies-Bouldin score calculates the tightness of a cluster by comparing the scattering of data within a cluster versus the distance between centroids of two clusters: the smaller the score, the more distinct are the clusters. We found that many wavelet frequencies with small Davies–Bouldin scores (Figure S2C, score ∼0.4) gave rise to clusters that correctly classify growth curves according to bacterial strains (Figure 3C, period = 24.6 h). This result suggests that the wavelet transform could filter and focus the data on informative frequencies by suppressing random fluctuations. We note that the use of raw growth curves resulted in the mis-identification of one strain (Figure 3D & Figure S2D). In addition, a bootstrap analysis shows that the wavelet-based method produces fewer numbers of misclassified strains when compared to using raw data (Figure S2E & F). These results further corroborate the effectiveness of the wavelet-based approach in strain identification.

Next, we hypothesized that strain classification can be enhanced by combining data measured in the presence of well-defined perturbations (Figure S3 & Text S1). Specifically, we perturbed bacterial growth by decreasing temperature, introducing a metabolic burden using a plasmid [35], or decreasing the nutrient concentration (Figure S3A & B, & Table S3). We concatenated growth curves of each bacterial strain into one time series (Figure S3C), which was used for strain identification. Indeed, the combination of the perturbation results improved strain identification by increasing the separation between clusters of MG1655z1 and BL21pro strains (Figure S3D). These analyses demonstrate that unbalanced growth environments can indeed improve the classification of bacterial strains.

**Figure 4:**
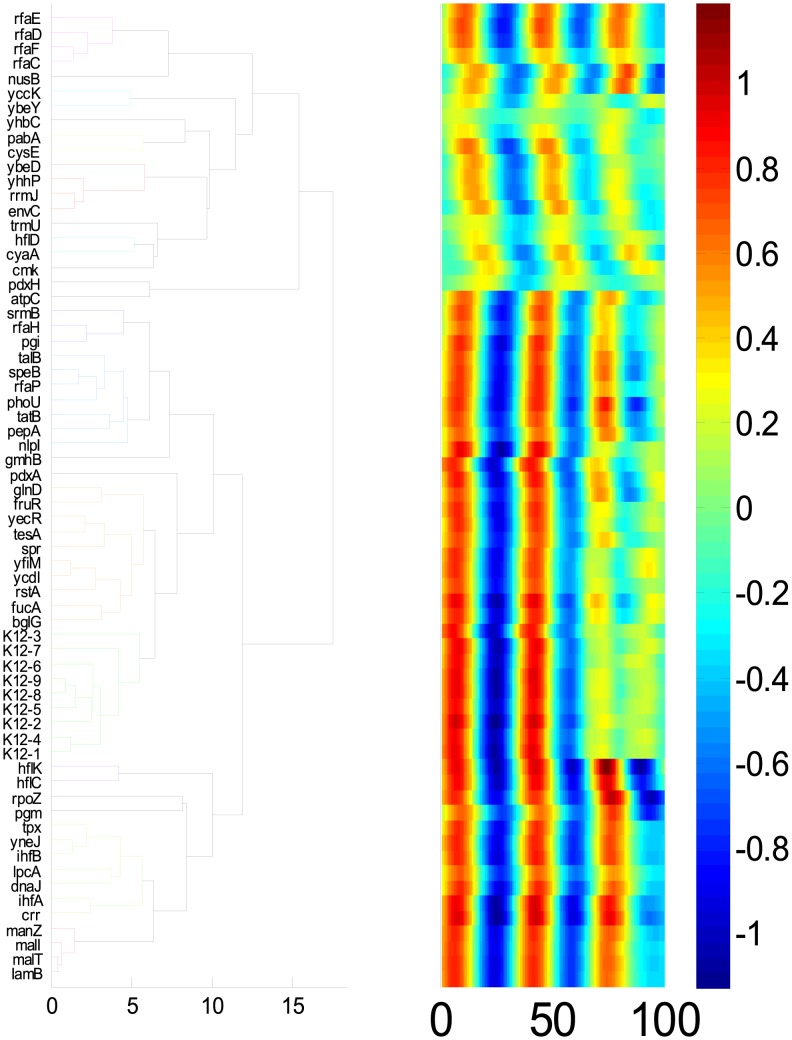
Analysis of bacteriophage lambda infection dynamics. Classification of bacterial knockout strains using unbalanced growth dynamics perturbed by the infection of bacteriophage lambda. Wild type K12 strains were classified into one tight cluster (left panel). Furthermore, two clusters associated with either lipopolysaccharide synthesis or LamB regulation were identified by distinct clusters. Each distinct cluster is represented by the same color in the tree. The right panel shows the corresponding phenotypic signatures of each strain.

In principle, our computational framework is applicable to the identification of reaction links between any system variables using cell-coupled signals. To test this, we analyzed growth curves of 66 *E. coli* knockout and wild-type strains under normal growth conditions and when perturbed by infection with bacteriophage lambda [36]. For each strain, we combined growth curves with and without bacteriophage lambda infection to enhance strain classification and then analyzed the data with our framework (Text S1). Our analysis gave rise to a tight cluster of K12 wild type strains (Figure 4). In addition, our framework clustered genes that are involved in *lamB* regulation (*lamB*, *malT*, *malI*,& *manZ*), which is involved in phage binding and transport [36]. Our framework also clustered genes involved in lipopolysaccharide synthesis (*rfaE*, *rfaD*, *rfaF*,& *rfaC*), which may be involved in phage transport [36]. We note that the two groups of genes could not be separated in a previous study that used raw growth curves without wavelet transform [36].

In addition to growth curves, our method can be applied to other system readouts with sufficient temporal fluctuations, such as gene expression. We first identified a rich dataset [37] that consists of high resolution of gene expression profiles from 1920 promoters in *E. coli* measured under six different conditions: glucose, no glucose, no amino acids, no phosphate, no nitrogen, and with ethanol. This dataset was used in the previous study to analyze promoter activities in different growth conditions. The authors found that expression levels of the translational machinery tightly follow growth rates. They proposed a model that accounts for resource distribution among promoters. In addition to the analysis of translational machinery, the dataset could also be used to identify correlations between promoters. Correlation between expression profiles is often used to infer regulatory strengths between transcription factors and DNA promoters [38], [39]. Here, we aimed to apply our wavelet-based approach to identify correlations between promoters using this dataset.

Since each growth condition in the dataset would represent a unique set of cell-coupled perturbations, we hypothesized that we could combine the data to estimate correlations between promoters. Each gene expression profile was transformed into the time-frequency domain. Next, we calculated the summation of wavelet coefficients at each frequency (*C_total,f_*) and time point (*C_total,t_*) (Figure S4A). We then identified the frequency and time point that exhibited the highest values of *C_total_*. Therefore, each gene expression profile is represented by only two features, which would speed up the following clustering analysis.

To facilitate the comparison of our results with literature data, we focused on promoters that are regulated by RpoH (identified using RegulonDB [40]), the master regulator of the heat shock response in *E. coli*. We chose this series of promoters because it is a well-studied pathway that protects bacteria from environmental stress [41]. Specifically, we asked if our wavelet-based method could identify RpoH-regulated promoters that are highly correlated using expression profiles of promoters across different growth conditions.

Indeed, our approach clustered promoters that are highly correlated with RpoH (Text S1, Figure S4). The classification provided a hierarchical view of the correlation strengths between promoters. For instance, expression profiles of both *lon* and *clpP* are highly correlated to that of *rpoH*. However, expression profiles of *dnaJ*, *dnaK*, and *clpX* are less correlated to that of *rpoH* (Figure S4B). These results suggest that the expression of *lon* and *clpP* might be regulated strongly by RpoH, while the expression of *dnaJ*, *dnaK*, and *clpX* might not be regulated solely by RpoH. Since our results are based solely on the correlation of expression profiles, we sought to evaluate our results with a previous study that analyzed binding strengths of RpoH to promoters using ChIP-on-chip assays [42]. Indeed, ten of the closely clustered promoters in our results exhibited high consensus sequence of RpoH binding sites (Figure S4C) when compared to the ChIP-on-chip assays. Therefore, our framework produces correlation strengths between promoters that are on par with more labor-intensive and expensive ChIP-on-chip methods.

**Figure 5:**
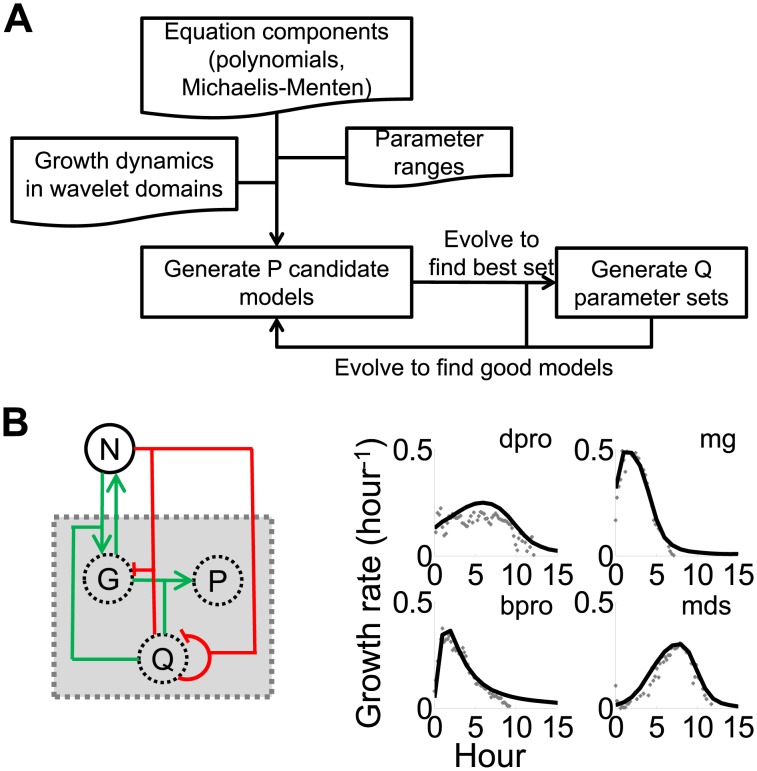
Reverse engineering of a growth model using the wavelet transform. A. The flowchart of a swarm algorithm that identifies growth models using the wavelet transform. The algorithm stochastically evolves growth models by combining different equation components and parameters. See detailed algorithm description in Text S1. B. An identified model (left panel) using unbalanced growth dynamics of four bacterial strains: MG1655z1 (mg), DH5αPro (dpro), BL21Pro (bpro), and MDS (mds) (right panel). The model can explain growth curves of the four bacterial strains with distinct growth dynamics. In the left panel, green lines represent activation while red lines represent repression. In the right panel, grey cross hairs represent original data. The black lines represent simulated data using the model.

Thus far, our analysis indicates that the growth rate fluctuations during unbalanced growth can effectively classify bacterial strains, growth conditions, and genes within the same pathways. Since our analyses suggest that cell-coupled signals can lead to better system identification (Figure 1 & 2), we hypothesized that growth rate fluctuations could be used to infer a minimal gene network that could account for dominant features of the growth dynamics. To test this hypothesis, we attempted to identify parsimonious growth models by using a swarm algorithm, which has been shown to converge rapidly towards global minimal solutions (Figure 5A & Text S1) [43]. A swarm algorithm is a stochastic optimization method inspired by natural evolution [44], [45]. It is based on heuristic search procedures that incorporate random variation and selection. Specifically, it simulates a social behavior where each individual in a swarm adjusts its evolution according to its own evolution experience and the evolution experience of other individuals [46]. The key to the success of this optimization strategy is information sharing between individuals to attain a common goal.

We adapted the swarm algorithm to evolve models by randomly selecting and combining basic equation components and kinetic parameters (Text S1). Specifically, we chose 12 basic equation components that consisted of either polynomials or Michaelis-Menten type equations, which cover commonly used formulations of reaction kinetics, including the power law, mass action, and enzymatic kinetics. The algorithm then created a population of bacterial growth models, each consisting of a specific combination of the equation components. For each population, the algorithm created individuals with randomly generated kinetic parameters. Next, the algorithm evaluated the individuals and identified the parameter set that gave rise to the best fit to the input data. The algorithm then evolved the other individuals based on the best parameter set. After a predefined number of iterations, the algorithm identified the best model and evolved the other models accordingly.

To constrain our search space, we made two simplifying assumptions. First, we assumed that bacterial growth was limited by a single substrate, which is consistent with the use of minimal growth media in our experiments. Second, we only considered a three-variable model, which can generate sufficiently complex dynamics to capture the dominant features of the experimental data.

Our search algorithm revealed a model that could fit all observed growth dynamics by appropriate parameterization (Equations 2–5). The average error per time-point is ∼0.01 hour^−1^, which is close to the readability value of our instrumentation suggesting a high goodness of fit.
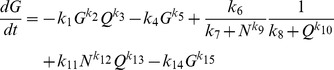
(2)

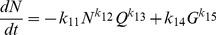
(3)

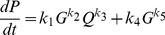
(4)

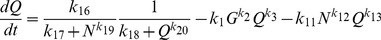
(5)where *G* represents a molecular species that modulates the growth rate, *N* represents the nutrient, *P* & *Q* represent two groups of molecular species, and *k*_i_ represents parameters that are optimized by the search algorithm. Parameter values of the optimized model are listed in Table S4. We assumed that node G directly modulates the growth rate with a proportionality constant of one.

This network consists of coupled positive and negative feedback loops (Figure 5B), which makes intuitive sense. Positive feedback loops can introduce a time-delay in the rise of the growth rate, but can then rapidly increase the growth rate after the time-delay. Negative feedback loops can maintain homeostasis of the growth rate. To further test the inferred model, we perturbed it by either increasing metabolic burden due to plasmid load or decreasing reaction rates due to low growth temperature. Specifically, plasmid load was simulated by increasing *k_4_* to 1.2 (Equation 2–5 & Table S4) and a lower growth temperature was simulated by reducing all of the kinetic constants by 30%. The perturbed model gave rise to results (Figure S5) that agree with the qualitative trends of our experimental results (Figure S3A). With plasmid load, the maximum growth rate is decreased and the entry time to stationary phase is maintained. With a lower growth temperature, the maximum growth rate is decreased and the entry time to stationary phase is extended.

These results show that unbalanced growth data can indeed be used to propose network motifs underlying growth dynamics. We note that our method could theoretically be extended to include more constraints and data that tend to improve model identification because the method is capable of handling multiple objectives and constraints.

## Discussion

In summary, our results demonstrate that unbalanced bacterial growth can provide rich information that facilitates network inference or classification of bacterial strains. The ability to distinguish microorganisms quickly is of critical importance in several fields including medicine, agriculture and biotechnology. Our approach relies on a simple growth assay and as such may offer a simpler and complimentary approach to current methods [5]. Current approaches of bacterial identification rely primarily on analysis of rRNA sequences that cannot differentiate between different strains of a species [47], [48]. Along this line, our method serves as an alternative that enables high throughput, strain-level phenotyping of bacteria. The ability of our framework to identify certain genes involved in the same cellular pathways may allow the identification of previously overlooked cellular components that contribute to the dynamics of a single network. Finally, our results may have value for improving experimental design in the study of cellular dynamics and gene regulation by allowing for more accurate and efficient construction of predictive models that are useful in suggesting hypotheses and exploring the likely effects of experimental interventions.

## Materials and Methods

For each bacterial strain, a single frozen stock was used to inoculate growth medium. We plated the frozen stock on LB agar plates and then randomly picked bacteria colonies for experiments. LB medium was used to prepare overnight cultures in 37°C. After ∼16 hours growth, the overnight cultures were diluted 100-fold again into fresh LB media to prepare the 2^nd^ batch of overnight cultures. The 2^nd^ batch of overnight cultures was diluted 100-fold into minimal M9 medium, supplemented with specific percentages of glucose and casamino acids (Table S3). Bacteria were grown in 200 µl of medium covered by 50 µl mineral oil (Sigma Aldrich, MO) in 96-well microplates at the specified temperature in a VICTOR 3 microplate reader (Perkin Elmer, MA). The microplates were shaken and measured at 10 minutes intervals. We did not observe significant media evaporation during the experimental duration. We also did not observe cross-contamination from neighboring wells. See Text S1 for additional details on Methods and Materials in this study.

## Supporting Information

Figure S1 **Minimal models for the comparison of information content.** A. A framework to measure parameter identifiability. For simplicity, we created models using linear flux equations. A model was transformed using Laplace transform, followed by calculation of its transfer function. The transfer function was used to calculate identifiability of each parameter, which yielded a correlation matrix between the parameters. Correlation factors close to either 1 or −1 indicate high correlation, hence low identifiability. For a sample model, four parameters were identifiable using a constant input (with fixed nutrient levels, red box). In contrast, all five parameters were identifiable using a cell-coupled input (blue box). The red line represents time series of a constant input signal. The blue line represents time series of a cell-coupled signal. B. Components of the minimal models. We constructed nonlinear models using flux reactions (green lines), positive regulation loops (blue lines), and negative regulation loops (red lines). We also included multiplicative reactions of two molecular species. C. An example of a model. Lines as described in (B). D. An estimated linear model (top panel, matrix A in Eq. S1) and the corresponding system dynamics (bottom panel). We estimated a linear model (top panel) using the resulting temporal dynamics from (B) (bottom panels). A blue line represents N. A green line represents P. A red line represents Q. A cyan line represents R.
(TIFF)

Figure S2 **Wavelet transform of bacterial growth rate curves.** A. Sample growth rate curves of seven bacterial strains. mg = MG1655z1, dpro = DH5αPro, pao = PAO1, mds = MDS42, bpro = BL21Pro, etec = ETEC, jm109 = JM109, top 10 = Top10. B. Wavelet transform of the bacterial strains. C. Davies-Bouldin scores at different wavelet periods. This metric was used to assess clustering quality. A lower score indicates better separation of clusters. D. Classification of growth rate curves into respective groups using the results from Figure 3C & D. The top panels show the classification results using raw growth data and the bottom panels show the classification results using wavelet transform. Red labels indicate growth curves that were mis-classified into the wrong groups. MG = MG1655z1, Dpro = DH5αPro, PAO = PAO1, MDS = MDS42, Bpro = BL21Pro, ETEC = ETEC, JM109 = JM109, Top10 = Top10. E. Histogram of misclassified strains using the wavelet-based method. A bootstrap method was used to remove one sample at a time for the clustering analysis. The wavelet method correctly classified all strains, except in one instance of the bootstrap samples. F. Histogram of misclassified strains using raw data. The clustering analysis classified all strains correctly in only one instance of the bootstrap samples.
(TIFF)

Figure S3 **Time series multiplexing for enhanced identification of bacterial strains.** A. Growth rates of MG1655z1 over time. MG1655z1 was subjected to five experimental perturbations: plasmid load (black line), lower incubation temperature (black dotted line), and lower nutrient (black dashed line). See Table S3 for detailed experimental setup. B. Growth rates of BL21Pro over time. BL21Pro was subjected to the same experimental perturbations as (A). C. Multiplex growth rates of MG1655z1. Growth curves in four different experimental conditions were multiplexed into one single growth curve. The multiplexed growth curve was used for strain identification in (D). D. Classification of BL21Pro and MG1655z1 using either the control or the multiplex growth curves. The multiplex growth curves significantly increased the separation between BL21Pro and MG1655z1, which suggests that they could be better identified in experiments.
(TIFF)

Figure S4 **Applying the computational framework to gene expression data.** A. Wavelet transforms of a time series of gene expression levels. Each expression profile was transformed into the wavelet domain, which gives rise to two wavelet features. The two wavelet features correspond to the time and period when the sum of wavelet coefficients is the highest, as indicated by the peak in the top and right panels. B. Classification of promoters that are regulated by RpoH across six experimental conditions [8]. Only a subset of promoters is classified together with *rpoH* (red color lines), suggesting that they share close dynamical similarity with RpoH. These promoters could be regulated more strongly by RpoH. A red arrow indicates the position of *rpoH*. The box on the right indicates the signature heatmap of each promoter. Each row of the signature heatmap represents the feature vector of each promoter. The feature vector consists of a concatenation of two features for each growth condition (from left to right: glucose, no glucose, no amino acids, no nitrogen, no phosphate, and with ethanol). C. Consensus sequences of −35 and −10 promoter regions for genes that cluster close (indicate by a * in panel B) or far (indicated by a # in panel B) from *rpoH*. In addition, we used high scoring and low scoring −35 and −10 consensus sequences from a ChIP-on-chip study of *rpoH* [6]. We compared these consensus sequences to the functional consensus sequence that was previously identified [5]. Changes in the functional consensus sequence have been shown to reduce transcription of downstream genes. Overall, we found that genes that clustered closer to *rpoH*, as well as those with high ChIP-on-chip scores, had a consensus sequence that was more similar to the functional consensus sequence than those that clustered farther away (and those with lower ChIP-on-chip scores).
(TIFF)

Figure S5 **Perturbation of the estimated growth model.** Predicted growth rates using the estimated model (Fig. 3B). To test the predictive power of the estimated growth model, we emulated either plasmid load or a lower growth temperature by modifying system parameters (Equation 2–5). To emulate plasmid load, *k_4_* was increased to 1.2 (Equation 2–5 & Table S4). To emulate a lower growth temperature, all kinetic constants were reduced by 30%. The predicted results agree qualitatively with our experimental results (Fig. S3A). Specifically, with plasmid load, maximum growth rates decrease, but the overall growth rate profile is similar between unperturbed and perturbed cells. With a lower growth temperature, maximum growth rates decrease and perturbed cells reach stationary phase later than the unperturbed cells.
(TIFF)

Table S1 **Genotypes of bacteria used in this study (** **Figure 3** **& Figure S2).** The detailed genotypes and sources of bacterial strains used in the study.
(DOCX)

Table S2 **Growth metrics extracted from bacterial growth curves (** **Figure 3** **).** We extracted three growth metrics from growth curves: maximum growth rate, final optical density (OD), and summation of differences. The indicated bacterial strains could not be distinguished by these metrics.
(DOCX)

Table S3 **Experimental conditions of each perturbation (Figure S3).** We used four culture conditions to perturb bacterial growth. Each culture condition perturbs one of three parameters: plasmid load, culture temperature, or nutrient concentration.
(DOCX)

Table S4 **Kinetic constants of** **Equations 2** **–** **5** **.** Parameters were identified using the swarm algorithm for the best fit of bacterial growth curves.
(DOCX)

Text S1 **The supporting information contains supporting methods, supporting results, Figures S1, S2, S3, S4, S5, and Tables S1, S2, S3, S4.**
(DOCX)
