# Supplementary figures and images for: Phenotypic Signatures Arising from Unbalanced Bacterial Growth
Source: PLoS Comput Biol. 2014 Aug 7;10(8):e1003751. doi: 10.1371/journal.pcbi.1003751 (PMC4125075; doi:10.1371/journal.pcbi.1003751)

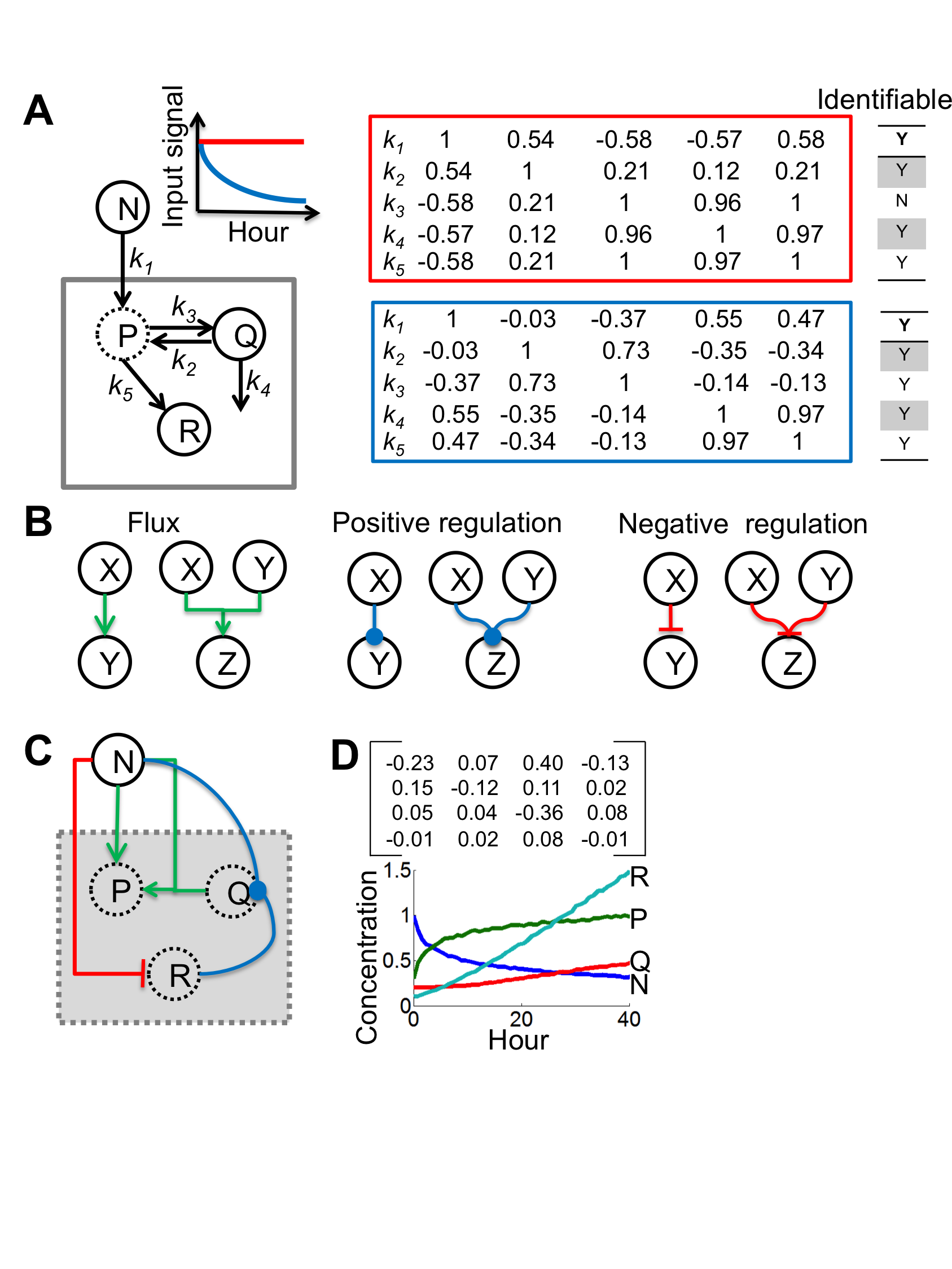

Supplement: Figure S1 — Minimal models for the comparison of information content. A. A framework to measure parameter identifiability. For simplicity, we created models using linear flux equations. A model was transformed using Laplace transform, followed by calculation of its transfer function. The transfer function was used to calculate identifiability of each parameter, which yielded a correlation matrix between the parameters. Correlation factors close to either 1 or −1 indicate high correlation, hence low identifiability. For a sample model, four parameters were identifiable using a constant input (with fixed nutrient levels, red box). In contrast, all five parameters were identifiable using a cell-coupled input (blue box). The red line represents time series of a constant input signal. The blue line represents time series of a cell-coupled signal. B. Components of the minimal models. We constructed nonlinear models using flux reactions (green lines), positive regulation loops (blue lines), and negative regulation loops (red lines). We also included multiplicative reactions of two molecular species. C. An example of a model. Lines as described in (B). D. An estimated linear model (top panel, matrix A in Eq. S1) and the corresponding system dynamics (bottom panel). We estimated a linear model (top panel) using the resulting temporal dynamics from (B) (bottom panels). A blue line represents N. A green line represents P. A red line represents Q. A cyan line represents R. (TIFF) [file pcbi.1003751.s001.tiff]

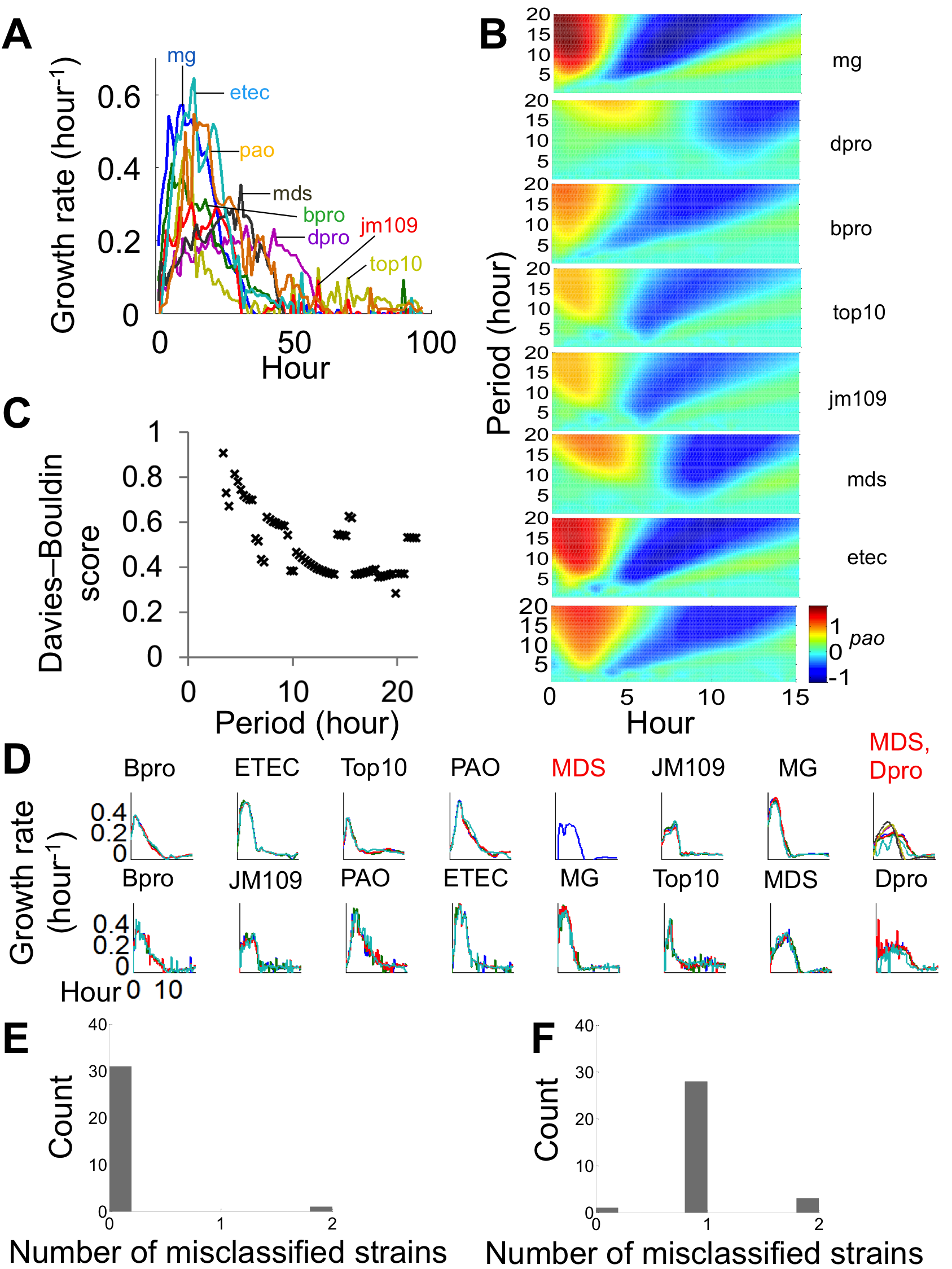

Supplement: Figure S2 — Wavelet transform of bacterial growth rate curves. A. Sample growth rate curves of seven bacterial strains. mg = MG1655z1, dpro = DH5αPro, pao = PAO1, mds = MDS42, bpro = BL21Pro, etec = ETEC, jm109 = JM109, top 10 = Top10. B. Wavelet transform of the bacterial strains. C. Davies-Bouldin scores at different wavelet periods. This metric was used to assess clustering quality. A lower score indicates better separation of clusters. D. Classification of growth rate curves into respective groups using the results from Figure 3C & D. The top panels show the classification results using raw growth data and the bottom panels show the classification results using wavelet transform. Red labels indicate growth curves that were mis-classified into the wrong groups. MG = MG1655z1, Dpro = DH5αPro, PAO = PAO1, MDS = MDS42, Bpro = BL21Pro, ETEC = ETEC, JM109 = JM109, Top10 = Top10. E. Histogram of misclassified strains using the wavelet-based method. A bootstrap method was used to remove one sample at a time for the clustering analysis. The wavelet method correctly classified all strains, except in one instance of the bootstrap samples. F. Histogram of misclassified strains using raw data. The clustering analysis classified all strains correctly in only one instance of the bootstrap samples. (TIFF) [file pcbi.1003751.s002.tiff]

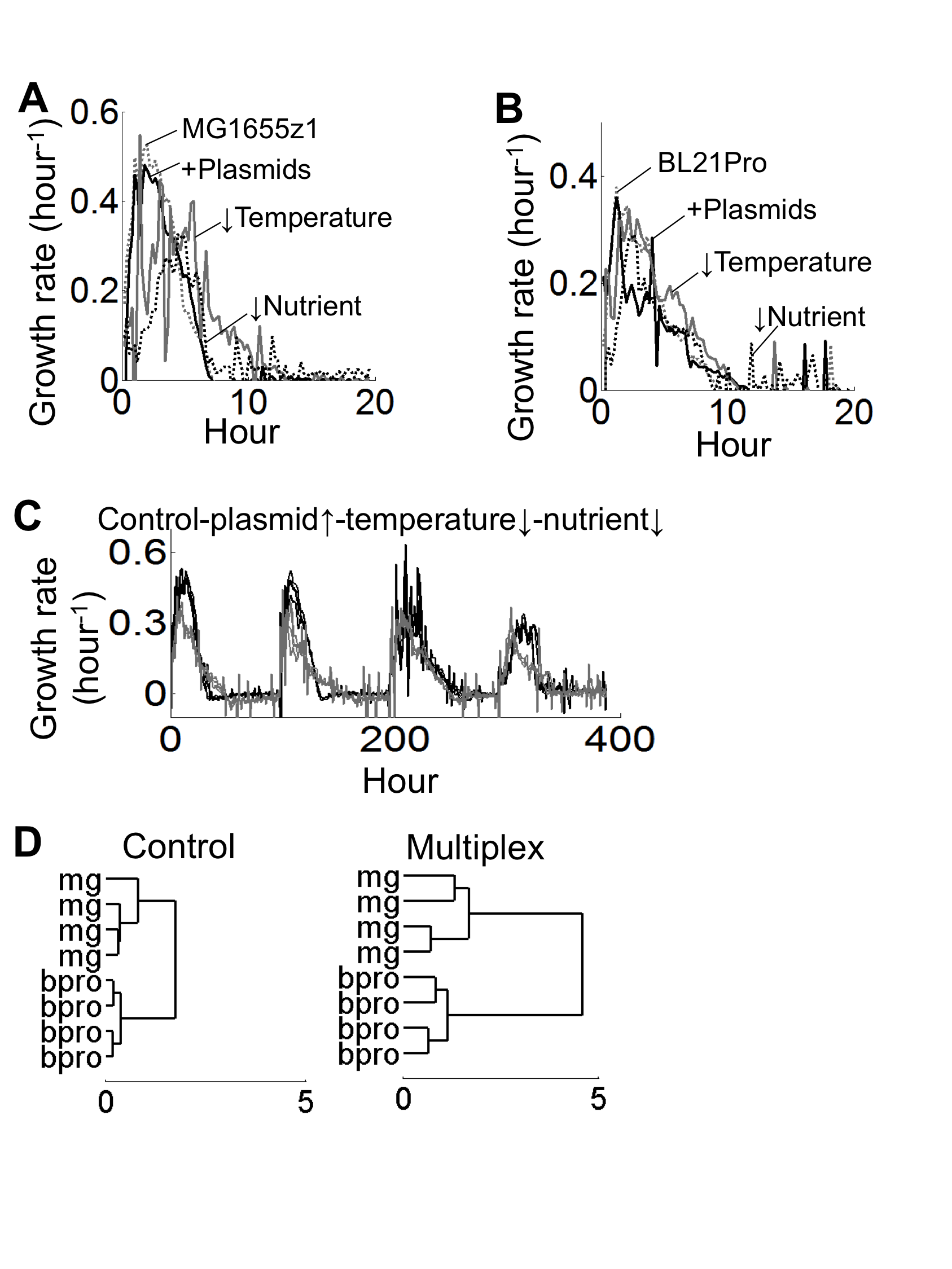

Supplement: Figure S3 — Time series multiplexing for enhanced identification of bacterial strains. A. Growth rates of MG1655z1 over time. MG1655z1 was subjected to five experimental perturbations: plasmid load (black line), lower incubation temperature (black dotted line), and lower nutrient (black dashed line). See Table S3 for detailed experimental setup. B. Growth rates of BL21Pro over time. BL21Pro was subjected to the same experimental perturbations as (A). C. Multiplex growth rates of MG1655z1. Growth curves in four different experimental conditions were multiplexed into one single growth curve. The multiplexed growth curve was used for strain identification in (D). D. Classification of BL21Pro and MG1655z1 using either the control or the multiplex growth curves. The multiplex growth curves significantly increased the separation between BL21Pro and MG1655z1, which suggests that they could be better identified in experiments. (TIFF) [file pcbi.1003751.s003.tiff]

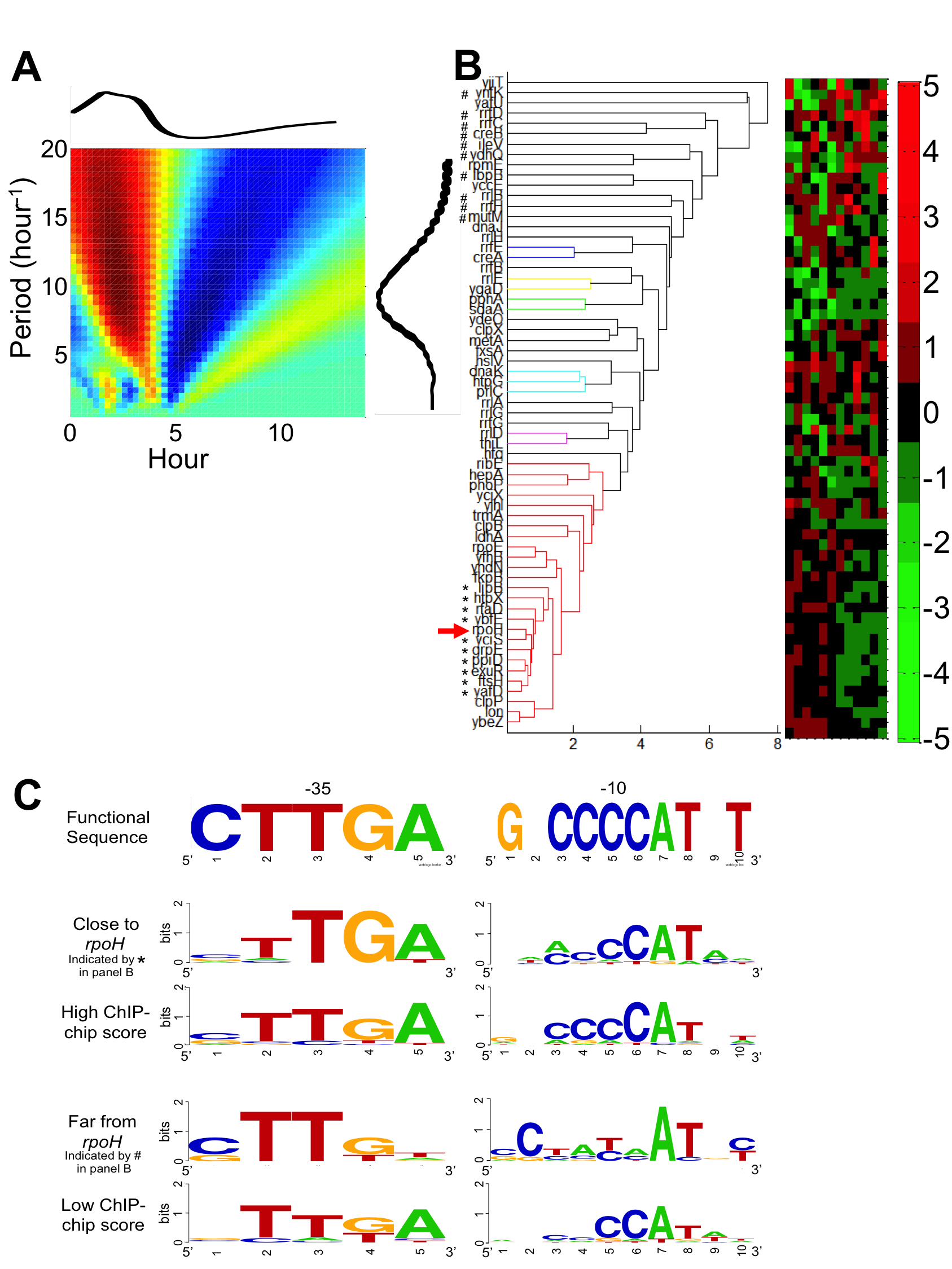

Supplement: Figure S4 — Applying the computational framework to gene expression data. A. Wavelet transforms of a time series of gene expression levels. Each expression profile was transformed into the wavelet domain, which gives rise to two wavelet features. The two wavelet features correspond to the time and period when the sum of wavelet coefficients is the highest, as indicated by the peak in the top and right panels. B. Classification of promoters that are regulated by RpoH across six experimental conditions [8]. Only a subset of promoters is classified together with rpoH (red color lines), suggesting that they share close dynamical similarity with RpoH. These promoters could be regulated more strongly by RpoH. A red arrow indicates the position of rpoH. The box on the right indicates the signature heatmap of each promoter. Each row of the signature heatmap represents the feature vector of each promoter. The feature vector consists of a concatenation of two features for each growth condition (from left to right: glucose, no glucose, no amino acids, no nitrogen, no phosphate, and with ethanol). C. Consensus sequences of −35 and −10 promoter regions for genes that cluster close (indicate by a * in panel B) or far (indicated by a # in panel B) from rpoH. In addition, we used high scoring and low scoring −35 and −10 consensus sequences from a ChIP-on-chip study of rpoH [6]. We compared these consensus sequences to the functional consensus sequence that was previously identified [5]. Changes in the functional consensus sequence have been shown to reduce transcription of downstream genes. Overall, we found that genes that clustered closer to rpoH, as well as those with high ChIP-on-chip scores, had a consensus sequence that was more similar to the functional consensus sequence than those that clustered farther away (and those with lower ChIP-on-chip scores). (TIFF) [file pcbi.1003751.s004.tiff]

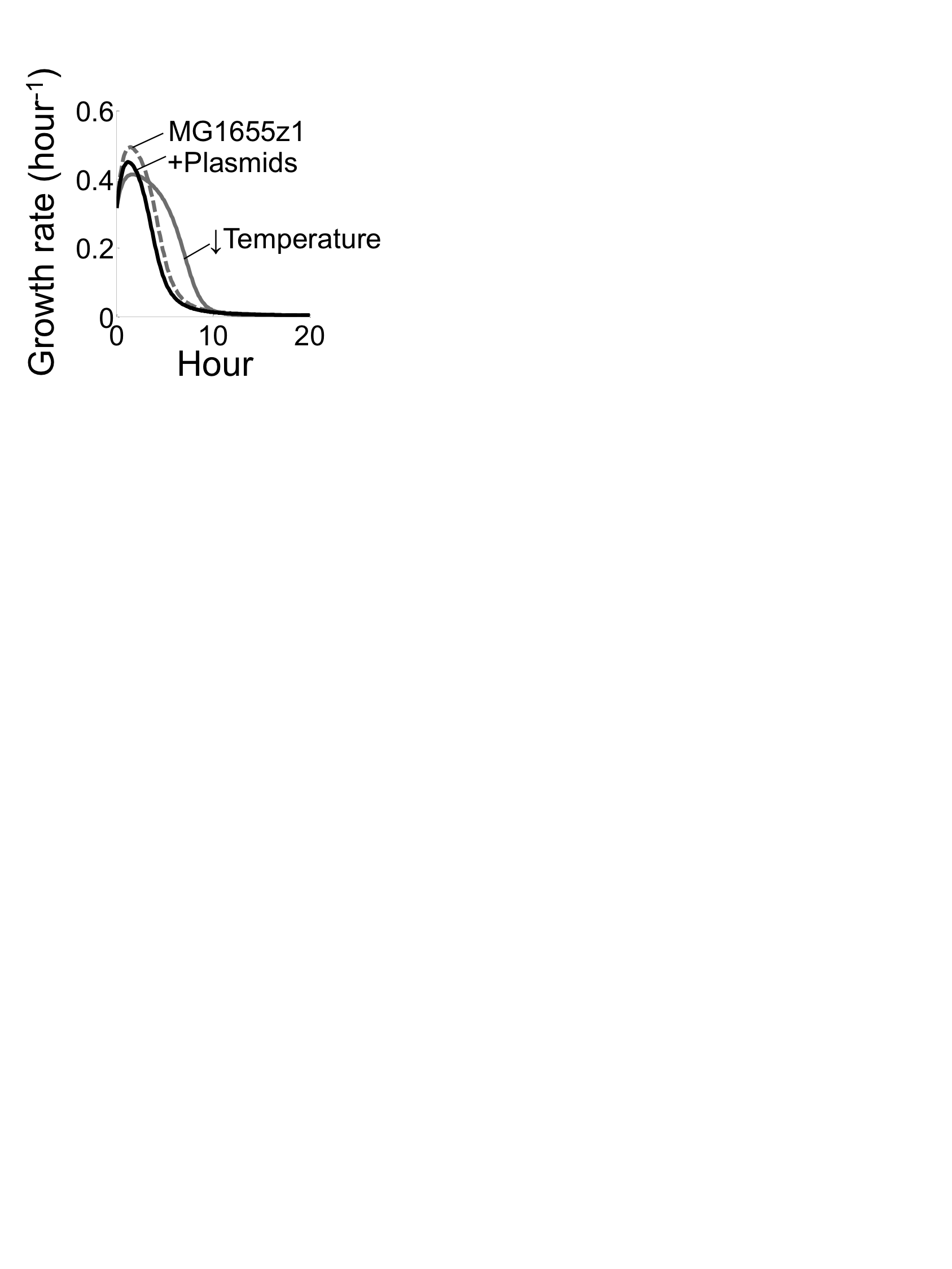

Supplement: Figure S5 — Perturbation of the estimated growth model. Predicted growth rates using the estimated model (Fig. 3B). To test the predictive power of the estimated growth model, we emulated either plasmid load or a lower growth temperature by modifying system parameters (Equation 2–5). To emulate plasmid load, k4 was increased to 1.2 (Equation 2–5 & Table S4). To emulate a lower growth temperature, all kinetic constants were reduced by 30%. The predicted results agree qualitatively with our experimental results (Fig. S3A). Specifically, with plasmid load, maximum growth rates decrease, but the overall growth rate profile is similar between unperturbed and perturbed cells. With a lower growth temperature, maximum growth rates decrease and perturbed cells reach stationary phase later than the unperturbed cells. (TIFF) [file pcbi.1003751.s005.tiff]
