# Supplementary material for: Phenotypic Signatures Arising from Unbalanced Bacterial Growth
Source: PLoS Comput Biol. 2014 Aug 7;10(8):e1003751. doi: 10.1371/journal.pcbi.1003751 (PMC4125075; doi:10.1371/journal.pcbi.1003751)
Supplement: Table S1 — Genotypes of bacteria used in this study ( Figure 3 & Figure S2). The detailed genotypes and sources of bacterial strains used in the study. (DOCX) [file pcbi.1003751.s006.docx]

| **Strain** | **Genotypes** | **Source** |
| --- | --- | --- |
| MG1655z1 | *F^-^ λ^–^ ilvG^-^ rfb-50 rph-1 Z1*(*LacR TetR SpR*) | * |
| BW25113 | F^-^ *λ^–^* Δ(*araD-araB*)*567* Δ*lacZ4787*(::rrnB-3) *rph-1* Δ(*rhaD-rhaB*)*568 hsdR514* | CGSC #7636 |
| Top10 | F^-^ *mcrA* Δ(*mrr-hsdRMS-mcrBC*) φ80*lac*ZΔM15 Δ*lacX74* *recA1* *araD139* Δ(*ara*-*leu*)7697 *galU* *galK* *rpsL* *endA1* *nupG* | Invitrogen |
| JM109 | F´*traD36* *proA^+^B^+^* *lacI^q^* Δ(*lacZ*)*M15*/ Δ(*lac-proAB*) *glnV44* *e14*^-^ *gyrA96 recA1 relA1 endA1 thi hsdR17* | NEB |
| BL21Pro | *F^–^ ompT hsdSB*(*rB^–^mB^–^*) *gal dcm* (containing *tetR, P_laci_^q^/laci*, and *Spr* on an autonomously replicating plasmid) | Clontech |
| MDS42 | Reduced genome based on MG1655 (3730 genes) [[2](#_ENREF_2)] | Scarab Genomics |
| DH5αPro | *F^–^ λ^–^ deoR endA1 gyrA96 hsdR17*(*rk^–^mk^+^*) *recA1 relA1 supE44 thi-1* Δ(*lacZYA-argF*)*U169* φ*80*δ*lacZ*Δ*M15 P_N25_/tetR P_laci_^q^/laci Spr* | Clontech |

* Elowitz MB, Leibler S (2000) A synthetic oscillatory network of transcriptional regulators. Nature 403: 335-338.
