# Supplementary material for: Phenotypic Signatures Arising from Unbalanced Bacterial Growth
Source: PLoS Comput Biol. 2014 Aug 7;10(8):e1003751. doi: 10.1371/journal.pcbi.1003751 (PMC4125075; doi:10.1371/journal.pcbi.1003751)
Supplement: Table S2 — Growth metrics extracted from bacterial growth curves ( Figure 3 ). We extracted three growth metrics from growth curves: maximum growth rate, final optical density (OD), and summation of differences. The indicated bacterial strains could not be distinguished by these metrics. (DOCX) [file pcbi.1003751.s007.docx]

|  | **Maximum growth rates (hour^-1^)** | **Final OD (Abs@600nm)** | **Summation of differences** |
| --- | --- | --- | --- |
| MG1655z1 | 0.497±0.018 | 0.424±0.012 | 0 |
| DH5αPro | 0.246±0.101 | 0.262±0.014 | 1.590±0.135 |
| BL21Pro | 0.378±0.014 | 0.210±0.005 | 0.757±0.048 |
| Top10 | 0.386±0.022 | 0.177±0.005 | 1.041±0.020 |
| JM109 | 0.265±0.023 | 0.161±0.008 | 0.986±0.081 |
| MDS42 | 0.288±0.016 | 0.166±0.012 | 1.333±0.093 |
| ETEC | 0.548±0.026 | 0.376±0.037 | 0.735±0.046 |
| PAO | 0.469±0.019 | 0.443±0.016 | 1.246 ±0.081 |

Note: For each column, numbers highlighted by the same color overlap within one standard deviation. For each strain, the standard deviation was calculated by using four replicates.
