# Supplementary material for: Phenotypic Signatures Arising from Unbalanced Bacterial Growth
Source: PLoS Comput Biol. 2014 Aug 7;10(8):e1003751. doi: 10.1371/journal.pcbi.1003751 (PMC4125075; doi:10.1371/journal.pcbi.1003751)
Supplement: Table S3 — Experimental conditions of each perturbation (Figure S3). We used four culture conditions to perturb bacterial growth. Each culture condition perturbs one of three parameters: plasmid load, culture temperature, or nutrient concentration. (DOCX) [file pcbi.1003751.s008.docx]

| **Perturbations** | **Culture conditions** | **Bacteria strain** |
| --- | --- | --- |
| Control | M9 minimal medium + 0.4% (w/v) glucose + 0.1% (w/v) casamino acids, 37^o^C | MG1655z1 or BL21Pro |
| +Plasmids | M9 minimal medium + 0.4% (w/v) glucose + 0.1% (w/v) casamino acids, 37^o^C | MG1655z1+**p15aTetCFP** or  BL21Pro+**p15aTetCFP** |
| ↓Temperature | M9 minimal medium + 0.4% (w/v) glucose + 0.1% (w/v) casamino acids, **30^o^C** | MG1655z1 or BL21Pro |
| ↓Nutrient | M9 minimal medium + **0.1% (w/v) glucose**, 37^o^C | MG1655z1 or BL21Pro |

Note: Bold letters represent growth perturbations as compared to the control.
