# Supplementary material for: Phenotypic Signatures Arising from Unbalanced Bacterial Growth
Source: PLoS Comput Biol. 2014 Aug 7;10(8):e1003751. doi: 10.1371/journal.pcbi.1003751 (PMC4125075; doi:10.1371/journal.pcbi.1003751)
Supplement: Table S4 — Kinetic constants of Equations 2 – 5 . Parameters were identified using the swarm algorithm for the best fit of bacterial growth curves. (DOCX) [file pcbi.1003751.s009.docx]

|  | dpro | mg | bpro | mds |
| --- | --- | --- | --- | --- |
| *k_1_* | 0.1415 | 1.8380 | 1.8491 | 1.6818 |
| *k_2_* | 2.1786 | 1.8417 | 2.1925 | 1.7195 |
| *k_3_* | 3.4245 | 2.6961 | 2.5492 | 2.2118 |
| *k_4_* | 3.1895 | 0.4204 | 0.7938 | 0.9846 |
| *k_5_* | 2.5454 | 2.6690 | 2.5310 | 3.1110 |
| *k_6_* | 0.2277 | 0.1862 | 0.0654 | 0.0301 |
| *k_7_* | 4.2904 | 1.0050 | 1.2625 | 3.8863 |
| *k_8_* | 0.5076 | 0.3195 | 1.4466 | 0.7548 |
| *k_9_* | 2.2438 | 1.6439 | 2.9155 | 2.1605 |
| *k_10_* | 2.2390 | 2.3842 | 2.8212 | 2.7691 |
| *k_11_* | 0.0499 | 1.2905 | 1.5960 | 1.9273 |
| *k_12_* | 1.4721 | 2.4837 | 2.6277 | 2.4872 |
| *k_13_* | 3.1517 | 2.5121 | 2.4699 | 3.4496 |
| *k_14_* | 0.1359 | 2.1153 | 0.2412 | 0.4012 |
| *k_15_* | 1.5035 | 3.2381 | 2.6511 | 2.3605 |
| *k_16_* | 1.6781 | 0.4921 | 1.4073 | 0.5961 |
| *k_17_* | 0.0773 | 0.1359 | 1.1564 | 0.0974 |
| *k_18_* | 5.1713 | 0.5549 | 0.7657 | 5.4746 |
| *k_19_* | 2.0444 | 2.6127 | 2.4453 | 3.4464 |
| *k_20_* | 2.6388 | 2.1413 | 2.4502 | 1.8081 |
