# Supplementary material for: Phenotypic Signatures Arising from Unbalanced Bacterial Growth
Source: PLoS Comput Biol. 2014 Aug 7;10(8):e1003751. doi: 10.1371/journal.pcbi.1003751 (PMC4125075; doi:10.1371/journal.pcbi.1003751)
Supplement: Text S1 — The supporting information contains supporting methods, supporting results, Figures S1, S2, S3, S4, S5, and Tables S1, S2, S3, S4. (DOCX) [file pcbi.1003751.s010.docx]

Text S1

**Phenotypic signatures arising from unbalanced bacterial growth**

Cheemeng Tan^1,2^, Robert Phillip Smith^3,5^, Ming-Chi Tsai^1^, Russell Schwartz^1,4^, Lingchong You^5,6*^

^1^Lane Center of Computational Biology, Carnegie Mellon University, Pittsburgh, PA 15213, USA

^2^Department of Biomedical Engineering, University of California Davis, Davis, CA 95616, USA

^3^Division of Mathematics, Science and Technology, Nova Southeastern University, Fort Lauderdale, FL 33314, USA

^4^Department of Biological Sciences, Carnegie Mellon University, Pittsburgh, PA 15213, USA

^5^Department of Biomedical Engineering, Duke University, Durham, NC 27708, USA

^6^Institute for Genome Sciences and Policy, Duke University, Durham, NC 27708, USA

* Correspondence and requests for materials should be addressed to Russell Schwartz (Email: [russells@andrew.cmu.edu](mailto:russells@andrew.cmu.edu)) and Lingchong You (E-mail: [you@duke.edu](mailto:you@duke.edu); Tel: 919-660-8408; Fax: 919-668-0795).

**Analysis of bacteriophage lambda data (Figure 4)**

The bacteriophage lambda data was obtained from a previous publication [[1](#_ENREF_1)]. For the clustering analysis, we first concatenated growth curves without bacteriophage lambda and with bacteriophage lambda infection. Next, we performed the same clustering analysis as above to separate the strains.

**Swarm algorithm (Figure 5)**

To generate our swarm algorithm, we modified an algorithm that was developed previously [[2](#_ENREF_2)]. The algorithm includes the following steps.

1. User Input: *N* equation components, parameter ranges, time series data, number of objective functions
2. Transform the time series data to time-frequency domains *W_in_* by wavelet transform
3. Initialize *P* population, each with a random subset of the equation components
4. Initialize *Q* individuals within each population. Each individual has a distinct parameter set
5. For each population and each objective function
6. Compute wavelet coefficients of all individuals, *W*
7. Compute differences *E* between *W* and *W_in_* at a specific frequency
8. Select a leader *L_1_* who has the smallest *E*
9. Cross each individual with *L_1_* to make (*Q*-1) new individuals
10. Repeat step (5) for *A* number of individual evolutions
11. Select the top *B* leaders *L_2_* who have the smallest *E* among the populations
12. Cross each population with leaders from the set *L_2_* to make (*P*-*B*) new populations
13. Stop if termination criteria is satisfied
14. )Proceed to step (5)

In step (2), we transformed the time series by using a continuous wavelet transform with a gaus4 wavelet. In step (3), each population had an equal probability of selecting a specific equation component. We also imposed a constraint that nutrient was consumed for growth, hence could only decrease in simulations. In step (8), each population had 0.25 probability of inheriting equation components from two parents, 0.25 probability of inheriting all but one equation components from one parent, 0.25 probability to add one equation component to the equation components of one parent, and 0.25 probability of creating a new set of equation components. We simulated an evolution by using 100 populations, 20 individuals, 20 individual evolutions, and 50 population evolutions.

**Multiplex data (Figure S3)**

We measured bacterial growth under different temperatures, different nutrient concentrations, or different metabolic burdens caused by the presence or absence of a plasmid (Figure S3 & Table S3). Next, we concatenated growth curves of each bacterial strain into one time series (Figure S3C) that was used for strain identification. Indeed, multiplexing of the perturbation results improved strain identification. A dendrogram shows the separation distance of two bacterial strains in the wavelet feature space (Figure S3D). With the multiplex data, the separation distance between the two groups of data is longer than that of the single data. This result suggests that with the multiplexed data, the MG1655z1 and BL21Pro strains could be separated more distinctly as compared with classification based on just the control growth data.

**Analysis of *rpoH* functional consensus sequences (Figure S4B & C)**

Previous studies have indicated that variations in the -35 and -10 sequences in the promoter region may affect transcription rate [[3](#_ENREF_3)] and the binding efficiency of regulatory proteins [[4](#_ENREF_4)]. To corroborate our analysis in Figure S4B, we examined if the -35 and -10 sequences of the promoters clustered closely to *rpoH* were more similar to the functional consensus sequence [[5](#_ENREF_5)] than those that clustered farther away from *rpoH*. We selected ten genes that clustered close to *rpoH* and ten genes that were farthest from *rpoH*. To complement this analysis, we also examined the -10 and -35 sequences of RpoH-regulated promoters identified in a previous ChIP-on-chip study [[6](#_ENREF_6)]. We used Weblogo to align the functional consensus sequences of genes that had the highest ChIP-on-chip scores (*dnaK, yciS, ybbN, ycjX,, rlmE, ybeZ, htpX, fxsA, hslV, clpP, ybeD, gapA*) and the lowest ChIP-on-chip scores (*glnS, hotC, hepA, yafD, idhA, yhdN, ydhQ, rpoD, sdaA, grpE, yghJ, ibpA, yceP, topA*).

We observed that promoters that clustered together with *rpoH* (our analysis) and those with high ChIP-on-chip scores [[6](#_ENREF_6)] had high consensus to that of the functional sequence. In contrast, promoters that clustered farther away from *rpoH* (our analysis) and those with low ChIP-on-chip scores had sequences that were dissimilar from the functional sequence. We note that this trend is more evident when comparing the -10 sequences. Therefore, our computational framework is capable of identifying promoters with sequences that are more similar to the functional consensus sequence. Our framework may serve as an alternative tool for ChIP-on-chip studies or serve as a less expensive, less labor intensive tool for analysis of gene regulation (Figure S4C).

**Plasmid construction**

The p15aTetCFP plasmid (p15a origin, kanamycin resistant) was constructed by inserting a polymerase chain reaction (PCR)-amplified CFP from the pCFPT7 plasmid into a pINV4 vector (Ron Weiss) downstream of the *P_Tet_* promoter [[7](#_ENREF_7)].

**References**

1. Maynard ND, Birch EW, Sanghvi JC, Chen L, Gutschow MV, et al. (2010) A forward-genetic screen and dynamic analysis of lambda phage host-dependencies reveals an extensive interaction network and a new anti-viral strategy. PLoS Genet 6: e1001017.

2. Tan C, Ray T, Tsai H (2003) A Comparative Analysis of Evolutionary Algorithm and Swarm Algorithm for Airfoil Design Problems. 41st Aerospace Sciences Meeting and Exhibit. Reno, Nevada.

3. Kosuri S, Goodman DB, Cambray G, Mutalik VK, Gao Y, et al. (2013) Composability of regulatory sequences controlling transcription and translation in Escherichia coli. Proc Natl Acad Sci 110:14024-9.

4. Xu J, McCabe BC, Koudelka GB (2001) Function-based selection and characterization of base-pair polymorphisms in a promoter of Escherichia coli RNA polymerase-70. J Bacteriol 183: 2866-2873.

5. Wang Y, deHaseth PL (2003) Sigma 32-dependent promoter activity in vivo: sequence determinants of the groE promoter. J Bacteriol 185: 5800-5806.

6. Wade JT, Roa DC, Grainger DC, Hurd D, Busby SJW, et al. (2006) Extensive functional overlap between [sigma] factors in Escherichia coli. Nat Struct Mol Biol 13: 806-814.

7. Lutz R, Bujard H (1997) Independent and tight regulation of transcriptional units in Escherichia coli via the lacR/O, the tetR/O and araC/I1-I2 regulatory elements. Nucleic Acids Res 25: 1203-1210.

8. Zaslaver A, Kaplan S, Bren A, Jinich A, Mayo A, et al. (2009) Invariant distribution of promoter activities in Escherichia coli. PLoS Comput Biol 5: e1000545.
